# Mechanism of Erastin-Induced Ferroptosis in MDA-MB-231 Human Breast Cancer Cells: Evidence for a Critical Role of Protein Disulfide Isomerase

**DOI:** 10.1128/mcb.00522-21

**Published:** 2022-05-02

**Authors:** Hongge Wang, Pan Wang, Bao Ting Zhu

**Affiliations:** a Shenzhen Key Laboratory of Steroid Drug Discovery and Development, School of Medicine, The Chinese University of Hong Kong, Shenzhen, China; b Shenzhen Bay Laboratory, Shenzhen, China; c School of Life Sciences, University of Science and Technology of China, Hefei, Anhui, China

**Keywords:** cell death, protein disulfide isomerase, lipid ROS, iNOS, ferroptosis, nitric oxide

## Abstract

Ferroptosis is a form of regulated cell death resulting predominantly from catastrophic accumulation of lipid reactive oxygen species (ROS). While the antioxidant systems that counter ferroptosis have been well characterized, the mechanism underlying ferroptosis-associated accumulation of lipid ROS remains unclear. In this study, we demonstrated that protein disulfide isomerase (PDI) is a novel mediator of ferroptosis, which is responsible for the accumulation of lipid ROS and ultimately ferroptosis in MDA-MB-231 human breast cancer cells. Treatment with erastin led to a significant increase in inducible nitric oxide synthase (iNOS)-mediated nitric oxide production, which contributes to the accumulation of the death-inducing cellular lipid ROS. Small interfering RNA (siRNA)-mediated PDI knockdown or pharmacological inhibition of PDI’s isomerase activity with cystamine strongly suppressed iNOS dimerization and its catalytic activation, subsequently prevented lipid ROS accumulation, and conferred strong protection against erastin-induced ferroptosis. Remarkably, PDI knockdown in MDA-MB-231 cells also largely abrogated the protective effect of cystamine against erastin-induced ferroptotic cell death. Together, these experimental observations demonstrate a noncanonical role of PDI in ferroptosis, which may serve as a potential therapeutic target for ferroptosis-related diseases.

## INTRODUCTION

Ferroptosis is a new form of cell death (1). Morphologically, ferroptosis is characterized by reduced mitochondrial volume, increased lipid bilayer membrane density, and reduction or disappearance of mitochondrial cristae, while the cell membrane appears to remain intact and the nucleus of normal size, with no overt chromatin condensation (2–5). Biochemically, there are characteristic intracellular changes involving glutathione (GSH) depletion and/or decreased glutathione peroxidase 4 (GPX4) activity, which then results in suppression of GPX4-mediated reduction of lipid peroxides (accompanied by accumulation of cellular lipid reactive oxygen species [ROS]) and, ultimately, an iron-dependent oxidative cell death (ferroptosis) (3, 4, 6, 7). At present, the precise molecular mechanism and signaling pathways by which the accumulation of cellular lipid ROS ultimately leads to ferroptotic cell death are still a matter of strong interest. The inability of cells to efficiently eradicate lipid ROS is thought to mostly result from cellular dysfunction in cysteine metabolism and transport, which ultimately leads to intracellular GSH depletion (8–10). Given that GSH is a reducing cosubstrate of GPX4, which is largely responsible for the clearance of lipid ROS, GSH depletion would result in GPX4 inactivation, lipid ROS accumulation, and ultimately, oxidative ferroptotic cell death (10–12).

The immortalized HT22 mouse hippocampal neuronal cell line, which lacks a glutamate receptor, has been widely used as an *in vitro* model for elucidating the mechanism of oxidative stress-induced neurotoxicity (13, 14). Earlier we had shown that the presence of high concentrations of extracellular glutamate could selectively deplete intracellular GSH in a time- and concentration-dependent manner in this neuronal cell line (15). Our results further showed that during glutamate-induced GSH depletion and cytotoxicity in HT22 cells, nitric oxide (NO) accumulation preceded O_2_^•−^ accumulation. While neuronal NOS (nNOS) in untreated HT22 cells existed mostly as a monomer, GSH depletion resulted in increased formation of the dimer nNOS in a time-dependent manner, which is also accompanied by time-dependent increase in its catalytic activity for NO formation (15, 16). Further, we found that nNOS dimerization was catalyzed by the isomerase activity of protein disulfide isomerase (PDI) (16). PDI is an important member of the thioredoxin superfamily (17, 18) and plays a critical role in catalyzing the oxidation and isomerization of disulfide bonds to facilitate nascent protein folding, conformational changes, and functional regulation (18–21). Our earlier study showed that cystamine, a small-molecule inhibitor of PDI’s isomerase activity, could effectively prevent GSH depletion-induced accumulation of NO and O_2_^•−^ as well as oxidative cytotoxicity in cultured HT22 cells (16).

It was reported earlier that MDA-MB-231 human breast cancer cells, which do not express estrogen receptors (ERs), are sensitive to erastin-induced ferroptotic cell death (22, 23). Using this MDA-MB-231 human breast cancer cell line as an *in vitro* model, the present study aims to investigate the mechanism of erastin-induced ferroptotic cell death, with a focus on assessing the role of PDI in the induction of ferroptosis. Our results show that PDI plays a critical role in mediating erastin-induced ferroptosis in ER-negative MDA-MB-231 human breast cancer cells.

## RESULTS

### iNOS protein and NO are induced in ferroptosis.

**FIG 1:**
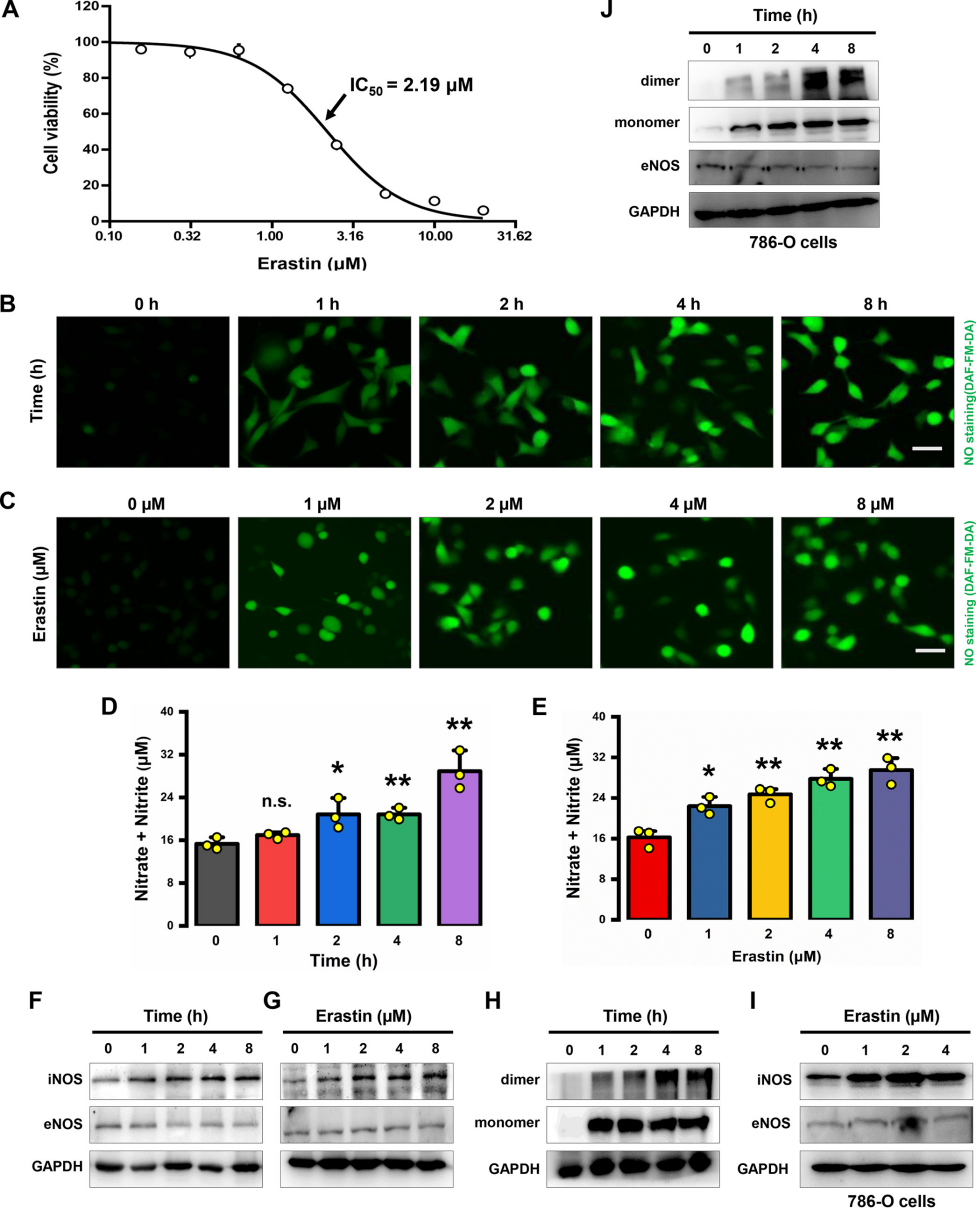
Erastin selectively increased iNOS protein levels. (A) Measurement of the IC_50_ value of erastin by MTT assay. Cell viability was assessed after the cells were exposed to erastin for 24 h. Data are shown as means ± SD (*n* = 5). (B, C) Representative images of cells stained with DAF-FM-DA. Increases in NO levels were induced in MDA-MB-231 cells by treatment with 5 μM erastin for the indicated time (B) or by treatment with 0, 1, 2, 4, or 8 μM erastin for 8 h (C), and then cells were stained and imaged with a fluorescence microscope. Scale bars = 50 μm. (D, E) After treatment of cells with erastin, as described for panels B and C, accumulation of nitrate and nitrite was measured using a Griess reagent. Data are shown as means ± SD (*n* = 3). *, *P* < 0.05, and **, *P* < 0.01, versus vehicle control. n.s., not significant. (F to J) Western blot analysis of iNOS and eNOS in MDA-MB-231 and 786-O cells treated with 5 and 2 μM erastin, respectively, for different lengths of time (F, H, J) or treated with different concentrations of erastin (as indicated) for 8 h (G, I). Following erastin treatment, cells were washed with ice-cold PBS and lysed by three cycles of freeze and thaw. Cytosolic fractions were prepared by centrifugation at 12,000 × *g* for 20 min, and Western blot analysis was performed using nondenaturing SDS-PAGE.

The constitutive NOSs, namely, neuronal NOS (nNOS [or NOSI]) and endothelial NOS (eNOS [or NOSIII]), typically produce smaller amounts of NO, whereas the inducible NOS (iNOS [or NOSII]) can produce large amounts of NO that can become highly cytotoxic (24, 25). Presently, it remains unknown whether NO is involved in ferroptosis-associated ROS accumulation. To determine the dose range for subsequent experiments, we first measured the 50% inhibitory concentration (IC_50_) value of erastin in MDA-MB-231 human breast cancer cells. After treatment of the cells with erastin for 24 h, cell viability was assessed by the 3-(4,5-dimethyl-2-thiazolyl)-2,5-diphenyl-2H-tetrazolium bromide (MTT) assay, and the IC_50_ value was found to be 2.2 μM in MDA-MB-231 cells (Fig. 1A). To evaluate the effect of erastin on NO production, we chose to measure the cellular NO levels using DAF-FM-DA (3-amino,4-aminomethyl-2′,7′-difluorescein) as a fluorescent probe that can react with NO. As expected, a rapid increase in cellular NO levels was observed in both a time- and dose-dependent manner following treatment of MDA-MB-231 cells with erastin (Fig. 1B and C). Similarly, a significant increase in nitrite levels was induced in a time- and dose-dependent manner in these cells following erastin exposure (Fig. 1D and E). It was noted that a significant increase in cellular NO level was evident at 1 h after erastin treatment, and NO reached its highest level at around 8 h. As increases in NOS protein level and particularly its dimer level would lead to increase in NO production, next we assessed iNOS and eNOS expression and their activity in MDA-MB-231 cells following erastin treatment. Remarkably, a strong increase in iNOS protein levels was observed in MDA-MB-231 cells, starting at 1 h after treatment with 5 μM erastin, and the increase continued in a time-dependent manner (Fig. 1F). Similarly, there was also a dose-dependent increase in iNOS protein level (Fig. 1G). In comparison, no similar change in protein levels was observed with eNOS (Fig. 1F and G). As iNOS is catalytically active only in its homodimer form, next we used nondenaturing SDS-PAGE coupled with Western blotting to probe the changes in iNOS dimer levels. We found that the levels of dimeric iNOS were markedly increased in a time-dependent manner after the cells were treated with 5 μM erastin (Fig. 1H), which was consistent with the time-dependent increase in NO production (Fig. 1B). To provide additional support for this conclusion, the experiment was also repeated in human renal clear cell carcinoma 786-O cells, which is a commonly used model cell line in ferroptosis-related studies. Consistent with the observations made in MDA-MB-231 cells, treatment of 786-O cells with 2 μM erastin also significantly increased iNOS protein levels and its dimerization in both a dose- and time-dependent manner (Fig. 1I and J). In contrast, the eNOS protein levels were not significantly affected (Fig. 1I and J).

### NO induces intracellular ROS accumulation.

**FIG 2:**
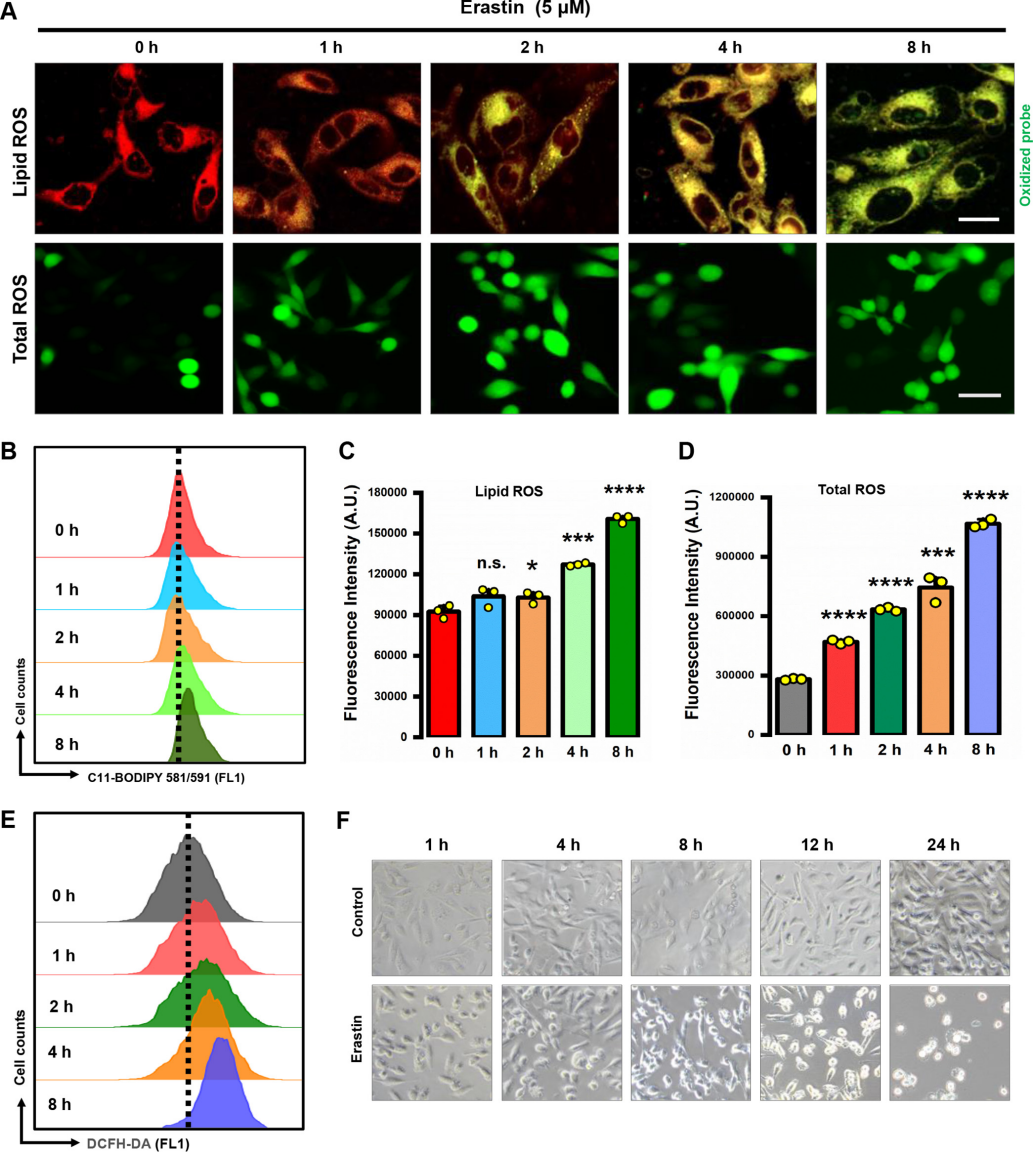
Erastin-induced cytosolic ROS accumulation. (A) Representative images of cells stained by C11-BODIPY and DCFH-DA. After treatment with 5 μM erastin for the indicated time, MDA-MB-231 cells were labeled with 2.5 μM C11-BODIPY or DCFH-DA prior to image analysis. The oxidized dye (in green) indicates lipid ROS. (Upper panel) Scale bars = 20 μm; (lower panel) scale bar = 50 μm. (B to E) Accumulation of lipid ROS (B, C) and cytosolic ROS (D, E) was assessed over time (0, 1, 2, 4, and 8 h) by flow cytometry following C11-BODIPY labeling or DCFH-DA labeling, respectively. A.U., absorbance units. Data are means ± SD (*n* = 3). **, *P* < 0.01; ***, *P* < 0.001; ****, *P* < 0.0001; n.s., not significant. (F) Time-dependent change in the gross morphology of cells treated with 5 μM erastin.

**FIG 3:**
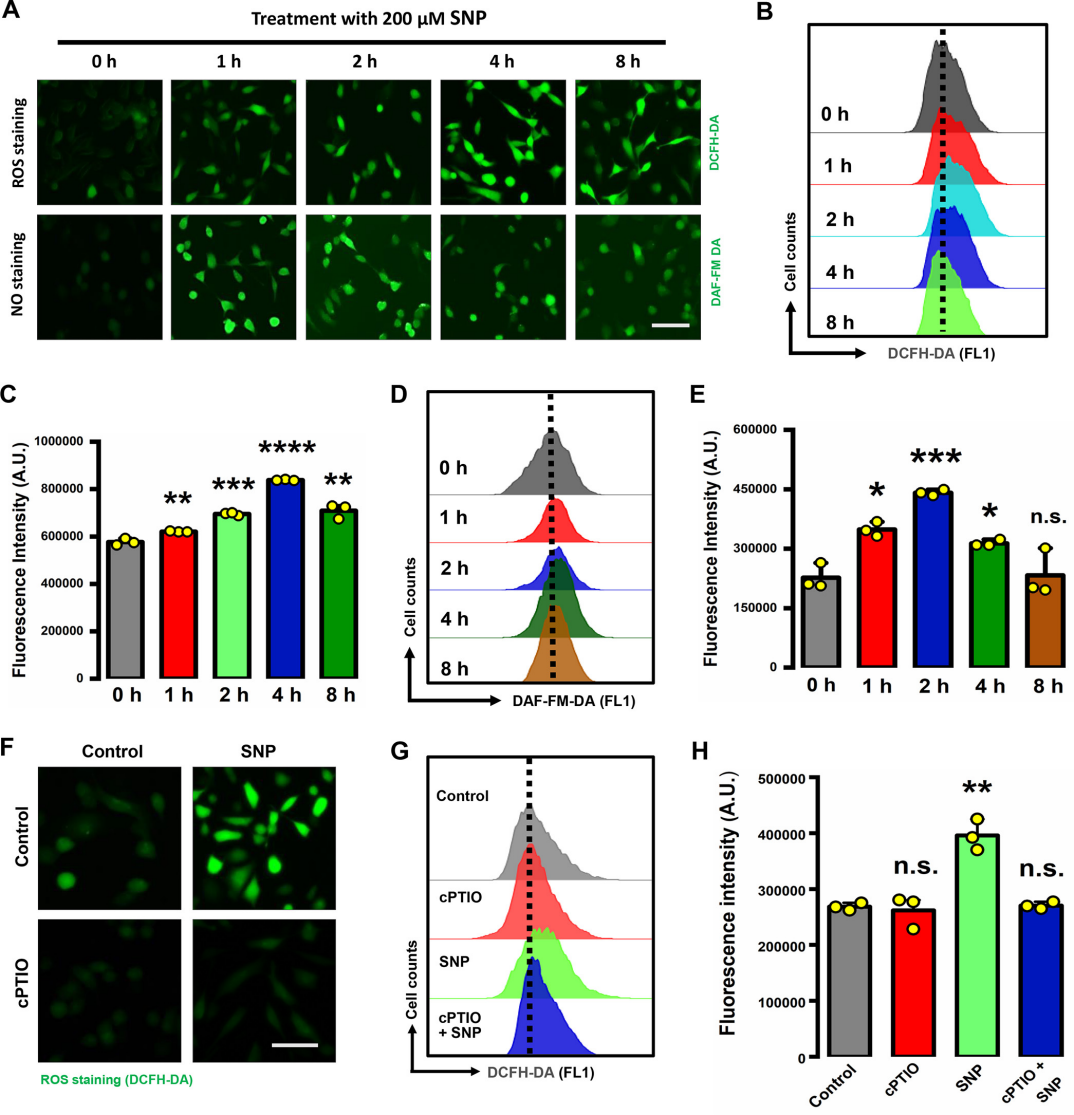
NO release directly promoted ROS accumulation. (A) Representative images of NO and ROS staining in MDA-MB-231 cells. Cells were treated with 200 μM SNP for the indicated time and then labeled with DCFH-DA (upper panel) or DAF-FM-DA (lower panel). Images were taken with a fluorescence microscope (*n* = 3). Scale bar = 50 μm. (B to E) After treatment as described for panel A, the accumulation of ROS and NO was determined following DCFH-DA (B, C) and DAF-FM-DA (D, E) labeling coupled with flow cytometry, respectively. (F) DCFH-DA labeling (for ROS) of MDA-MB-231 cells treated with 200 μM SNP ± 50 μM cPTIO for 8 h. Cells were stained by DCFH-DA, and images were taken with a fluorescence microscope (*n* = 3). Scale bar = 50 μm. (G, H) After treatment as for panel F, cytosolic NO production was assessed by flow cytometry following DCFH-DA labeling. Data are means ± SD (*n* = 3). *, *P* < 0.05; **, *P* < 0.01; ***, *P* < 0.001; ****, *P* < 0.0001; n.s., not significant.

In addition to our observation that erastin could induce a rapid and progressive accumulation of NO, analysis by fluorescence microscopy (with the aid of the fluorescent probes DCFH-DA [2′,7′-dichlorodihydrofluorescein diacetate] and C11-BODIPY) further showed that treatment of MDA-MB-231 cells with erastin (5 μM) caused a time-dependent increase in total cellular and lipid ROS levels, starting as early as 1 h after erastin exposure (Fig. 2A). Quantitative analysis by flow cytometry confirmed the observations of fluorescence microscopy (Fig. 2B to E). A pronounced increase in the accumulation of lipid ROS preceded cellular detachment and overt death (Fig. 2A), and the former occurred starting at approximately 8 h after erastin exposure (Fig. 2F). To provide further support for the role of NO in initiating ROS accumulation, we next chose to test the dose- and time-dependent effects of sodium nitroprusside (SNP), which is known to directly release NO, on ROS accumulation. The results showed that the amount of ROS accumulation was markedly increased after a 1-h treatment with 200 μM SNP and reached high levels at 8 h after SNP treatment (Fig. 3A to C), as assayed by flow cytometry using the fluorescent probe DCFH-DA. It was observed that the NO levels started to gradually decrease at 2 h after SNP exposure (Fig. 3A, D, and E). In addition, the SNP-induced ROS accumulation could be effectively inhibited by cPTIO [2-(4-carboxyphenyl)-4,5-dihydro-4,4,5,5-tetramethyl-1H-imadazol-1-yloxy-3-oxide], a specific NO scavenger (Fig. 3F to H). Together, these observations demonstrate that elevations in cellular NO levels can result in a time-dependent ROS accumulation, which peaks at ~8 h following NO increase.

### NO accumulation leads to ferroptosis.

**FIG 4:**
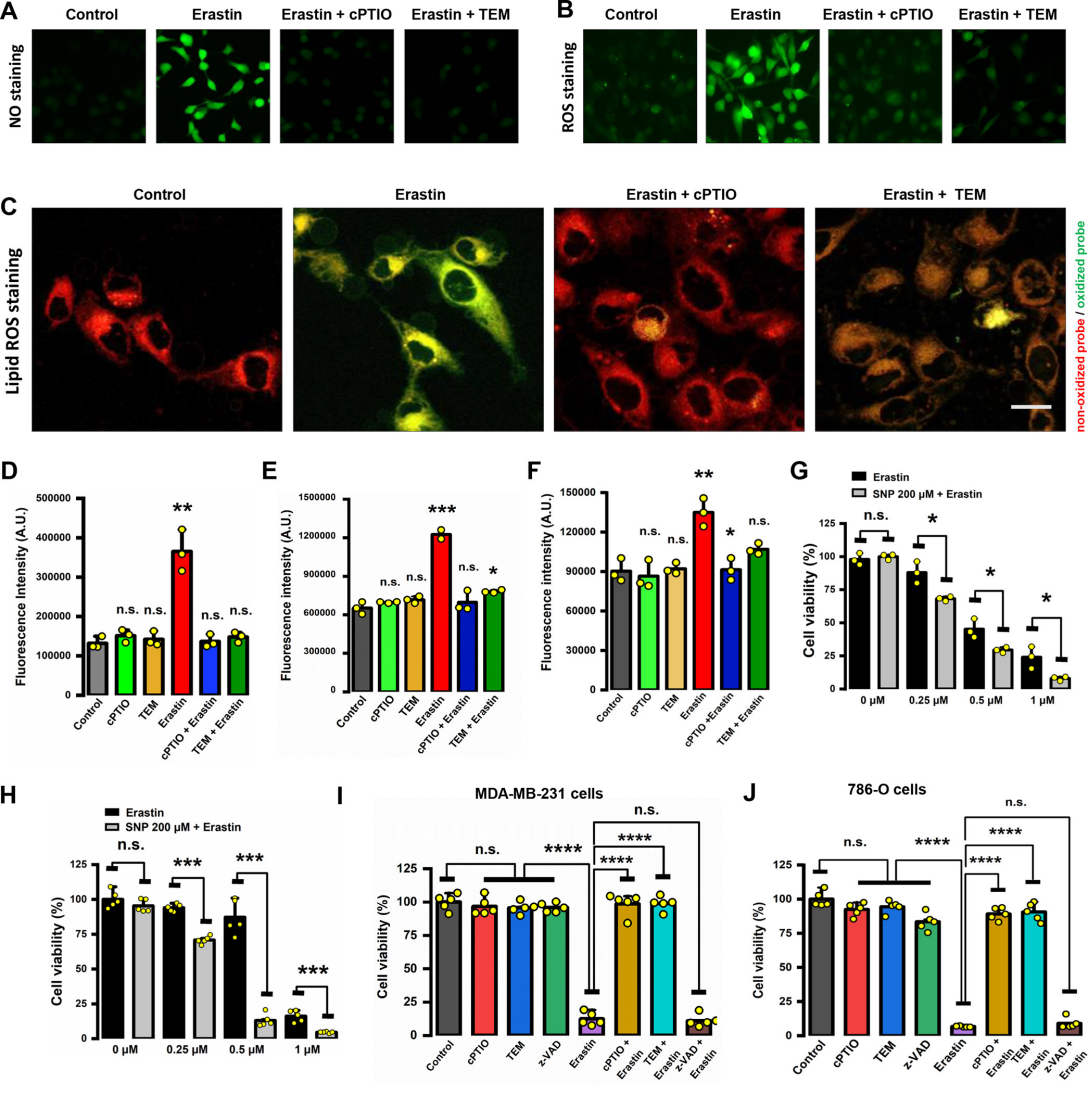
NO was a major source of ROS during erastin-induced ferroptosis. (A to C) Abrogation of erastin-induced ROS accumulation by NO scavengers. Cells were treated with 5 μM erastin ± 50 μM cPTIO or 100 μM TEM for 8 h, and then cells were stained with DCFH-DA (A), DAF-FM-DA (B), and C11-BODIPY (C). Images were taken with a fluorescence microscope (*n* = 3). Scale bar = 25 μm. (D to F) Accumulation of NO, cytosolic ROS, and lipid ROS was determined following dye staining coupled with flow cytometry. Data are means ± SD (*n* = 3). *, *P* < 0.05; **, *P* < 0.01; ***, *P* < 0.001; n.s., not significant. (G, H) SNP increased the sensitivity of cells to erastin-induced cytotoxicity. MDA-MB-231 (G) and 786-O (H) cells were treated with 200 μM SNP ± erastin for 24 h, and then cell viability was assessed by MTT assay. (I, J) Ferroptotic cell death was partially rescued by NO scavengers, but not by z-VAD-FMK (an apoptosis inhibitor). Cells were treated with 5 μM erastin ± 50 μM cPTIO, 100 μM TEM, or 2.5 μM z-VAD-FMK for 24 h, and then cell viability was assessed by MTT assay. Data are means ± SD (*n* = 5). *, *P* < 0.05; **, *P* < 0.01; ***, *P* < 0.001; ****, *P* < 0.0001; n.s., not significant.

Next, we sought to determine whether an increase in cellular NO levels contributes to chemically induced ferroptosis. First, we determined the effect of cPTIO on erastin-induced cell death in MDA-MB-231 cells. We found that treatment of the cells with 50 μM cPTIO caused a strong reduction in NO levels (Fig. 4A and D), which was accompanied by progressive reductions in total ROS level (Fig. 4B and E) and ferroptosis-associated lipid ROS level (Fig. 4C and F). Similarly, the prolonged period of either NO production or lipid ROS accumulation was prevented by treating cells with 2,2,6,6-tetramethyl-1-piperidinyloxy (TEM), which has both NO- and O_2_^−^-scavenging activities (Fig. 4A and C). Importantly, our data showed that SNP sensitized both MDA-MB-231 (Fig. 4G) and 786-O (Fig. 4H) cells to erastin’s cytotoxicity, suggesting that higher cellular levels of NO can enhance erastin-induced ferroptosis. Moreover, treatment of the cells with cPTIO, but not z-VAD-FMK, strongly abrogated erastin-induced ferroptosis in these cells (Fig. 4I and J), arguing against the involvement of apoptosis. Consistent with the observation that NO scavenger cPTIO has a protective effect against erastin-induced cell death, we also found that treatment of cells with TEM strongly reduced erastin-induced cytotoxicity (Fig. 4I and J). Together, these data suggest that the iNOS-mediated NO production (which is followed by cellular ROS accumulation) is an important initial step in erastin-induced ferroptotic cell death.

### iNOS is involved in ferroptosis.

**FIG 5:**
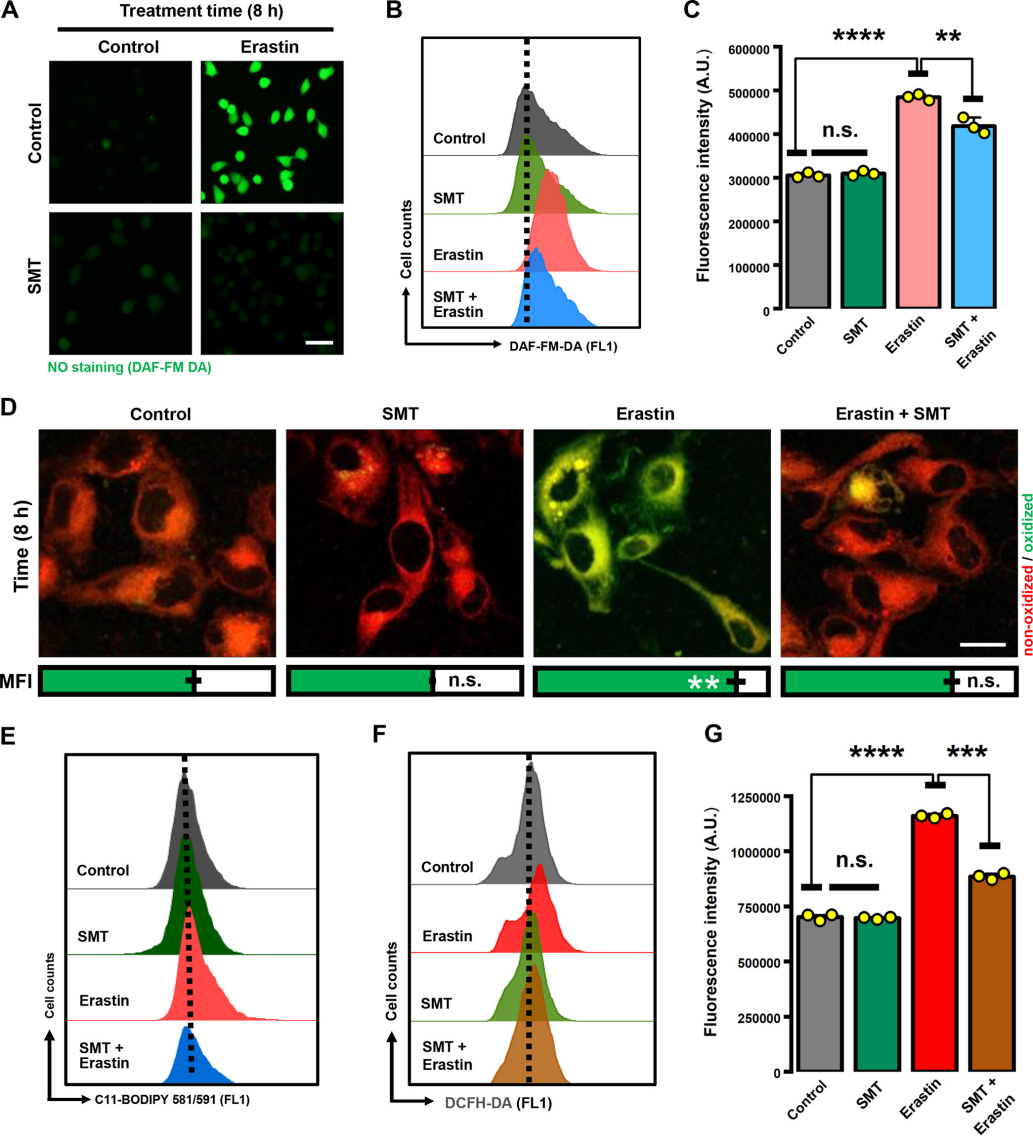
Lipid ROS accumulation was dependent on iNOS activity. (A) Representative images of NO staining in MDA-MB-231 cells. Cells were treated with 5 μM erastin ± 100 μM SMT (an iNOS inhibitor) for 8 h and then labeled with DAF-FM-DA. Images were taken with a fluorescence microscope (*n* = 3). Scale bar = 45 μm. (B, C) After treatment as in panel A, cytosolic NO production was assessed by flow cytometry following DAF-FM-DA labeling. (D to G) Attenuation of ROS accumulation by iNOS inhibitor SMT. After MDA-MB-231 cells were treated with 5 μM erastin ± 100 μM SMT for 8 h, confocal images of cells following C11-BODIPY labeling were taken (D), and lipid ROS accumulation was assessed by flow cytometry (E). MFI, mean fluorescence intensity (A.U.). Scale bar = 25 μm. Total ROS was determined following DCFH-DA labeling coupled with flow cytometry (F, G). Data are means ± SD (*n* = 3). **, *P* < 0.01; ***, *P* < 0.001; ****, *P* < 0.0001; n.s., not significant.

**FIG 6:**
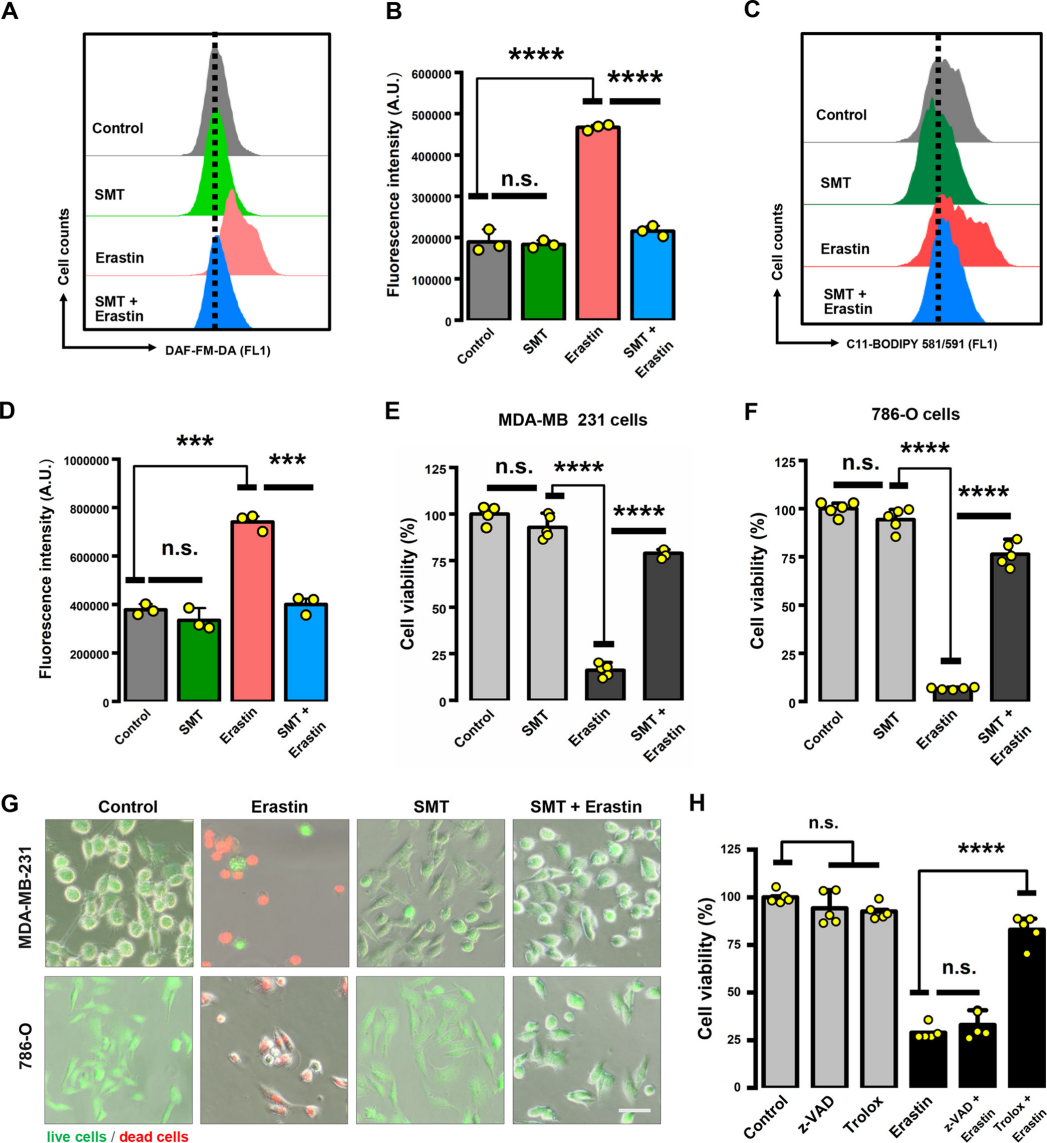
iNOS activity contributed to erastin-induced ferroptosis. (A to D) After 786-O cells were treated with 2 μM erastin ± 100 μM SMT for 8 h, cytosolic NO production (A, B) and lipid ROS accumulation (C, D) were assessed by flow cytometry following labeling with DAF-FM-DA or C11-BODIPY. Data are means ± SD (*n* = 3). ***, *P* < 0.001; ****, *P* < 0.0001; n.s., not significant. (E to G) Inhibition of iNOS activity protected cells from erastin-induced cell death. MDA-MB-231 and 786-O cells were treated with 5 μM (E) or 2 μM (F) erastin ± 100 μM SMT for 24 h. Cell viability is shown as a percentage of that of the vehicle control group. (G) Live/dead (green/red) fluorescent images of cells following calcein AM-PI staining. Cells were stained after exposure to 5 μM or 2 μM erastin ± 100 μM SMT for 24 h. Scale bar = 40 μm. (H) Cell viability change. MDA-MB-231 cells were treated with Trolox (50 μM) or z-VAD-FMK (2.5 μM) alone or in combination with erastin (5 μM) for 24 h. Data in panels E, F, and H are means ± SD (*n* = 5). ****, *P* < 0.0001; n.s., not significant.

**FIG 7:**
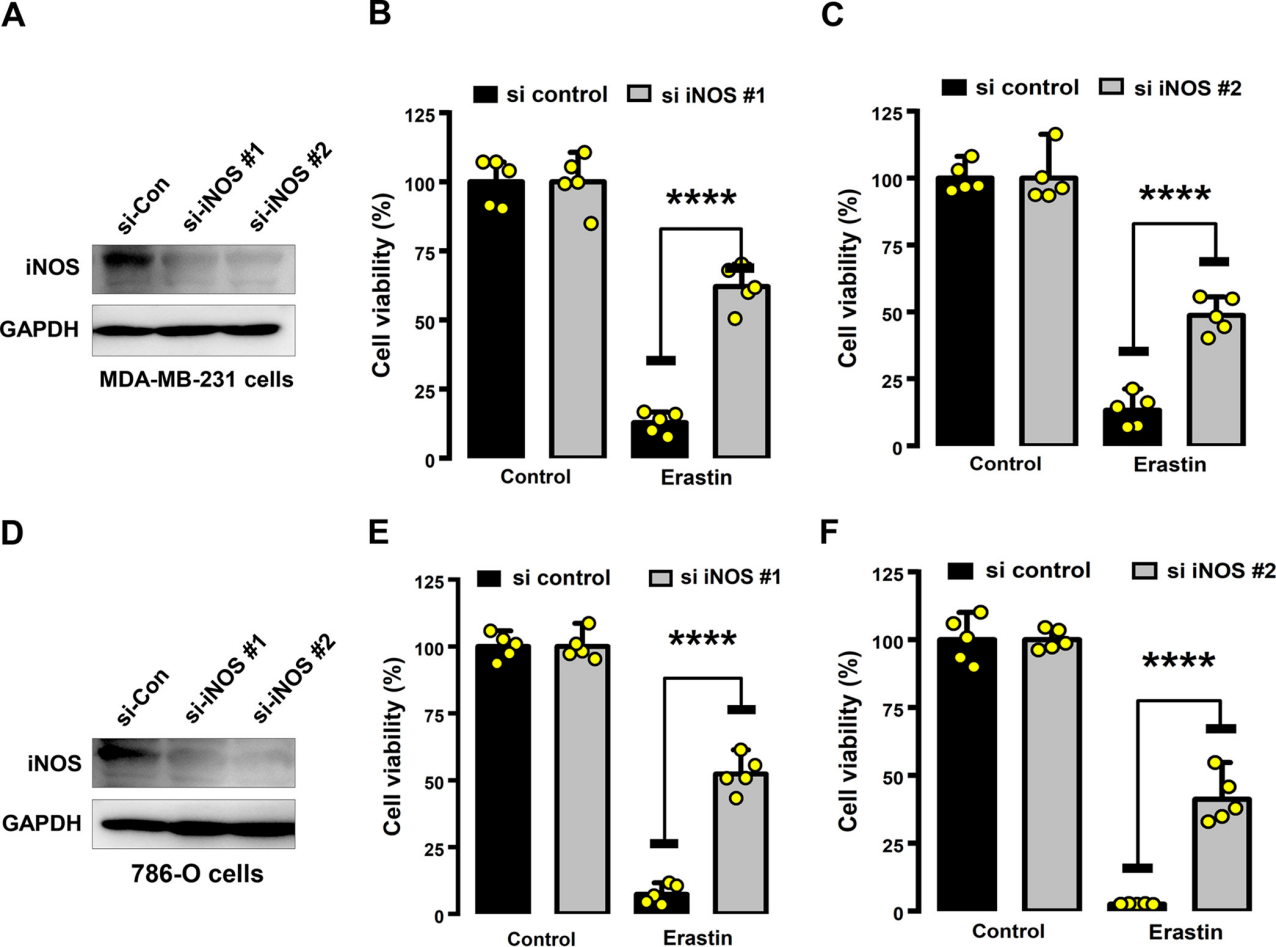
iNOS knockdown protected cells against erastin-induced cell death. (A, D) Confirmation by Western blotting of iNOS knockdown by siRNAs. Cells were transfected with two individual iNOS siRNAs using the RNAiMAX reagent for 24 h, and then Western blot analysis was performed to determine the knockdown efficiency in MDA-MB-231 cells (A) and 786-O cells (D). (B, C, E, F) iNOS knockdown by siRNAs significantly suppressed erastin-induced cell death. After siRNA transfection for 24 h, cells were treated with erastin for another 24 h. Cell viability of MDA-MB-231 cells (B, C) and 786-O cells (E, F) was measured with the MTT assay and is shown as a percentage of that of the vehicle control group. Data are means ± SD (*n* = 5). ****, *P* < 0.0001; n.s., not significant.

Based on these observations, we hypothesized that the iNOS-NO pathway may contribute to ROS accumulation, which subsequently causes ferroptotic cell death. To test this hypothesis, we first treated the cells with a known iNOS inhibitor, *S*-methyl-isothiourea (SMT), and found that SMT could effectively block the production of cellular NO (Fig. 5A to C), lipid ROS (Fig. 5D and E), and total ROS (Fig. 5F and G) in MDA-MB-231 cells. A similarly strong reduction in NO and lipid ROS levels was also observed in 786-O cells by joint treatment with SMT (Fig. 6A to D). Importantly, SMT also exerted a strong protection against erastin-induced cell death in both MDA-MB-231 cells (Fig. 6E and G) and 786-O cells (Fig. 6F and G), suggesting that elevated cellular NO levels in these cells are critically involved in erastin-induced cytotoxicity. The erastin-induced cell death was blocked by joint treatment of the cells with Trolox (6-hydroxy-2,5,7,8-tetramethylchroman-2-carboxylic acid [an antioxidant and ferroptosis inhibitor]), whereas z-VAD-FMK (a pan-caspase inhibitor) did not exert a meaningful protection (Fig. 6H). To provide further evidence that iNOS is involved in erastin-induced ferroptosis, two different small interfering RNA (siRNA) sequences were designed to target iNOS, and a nontargeting random siRNA sequence was used as a negative control. Consistent with the observations with SMT, iNOS knockdown conferred a strong protection against erastin-induced cell death in both MDA-MB-231 (Fig. 7A to C) and 786-O cells (Fig. 7D to F). Together, these results suggest that erastin-induced ROS accumulation and ferroptosis likely result from an increase in iNOS-mediated NO production and thus further substantiate an important role of the iNOS-NO pathway in ferroptosis.

### PDI catalyzes iNOS dimerization.

**FIG 8:**
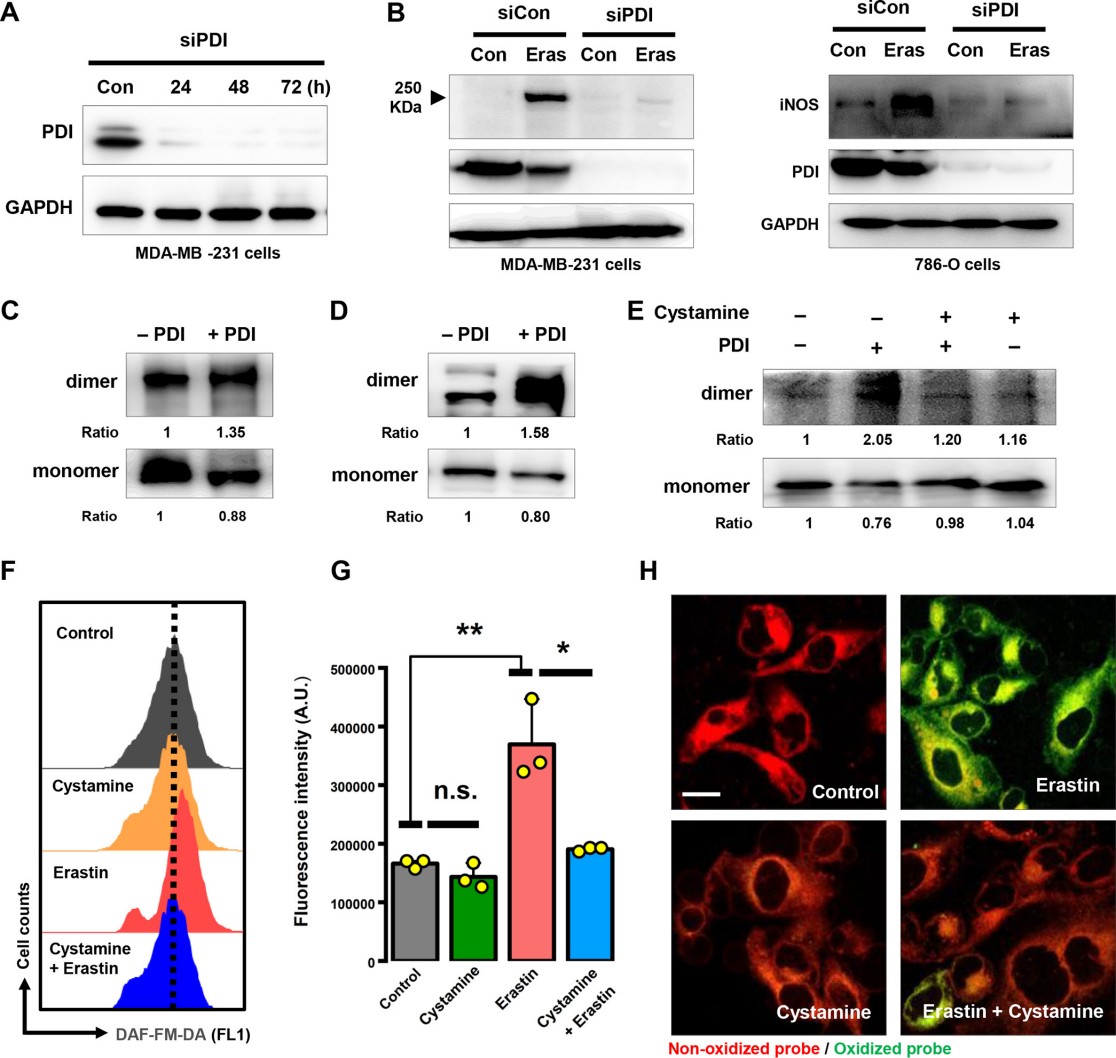
PDI was involved in iNOS dimerization. (A) Confirmation by Western blotting of PDI knockdown by siRNAs. Three individual PDI siRNAs were mixed and transfected at a working concentration of 100 nM with the RNAiMAX reagent. (B) PDI knockdown by siRNAs (48 h) significantly suppressed iNOS dimerization in MDA-MB-231 and 786-O cells treated with erastin. (C) Lysates from iNOS-overexpressing HEK-293 cells were incubated with or without purified PDI protein (1 mg/mL) at 4°C for 1 h, and then the dimer and monomer forms of iNOS were determined by nondenaturing SDS-PAGE (with 30 μg protein in each lane). (D, E) Dimer and monomer forms of iNOS were detected after incubation of purified iNOS protein (5 μg) with or without PDI (1 mg/mL) at 4°C for 1 h (C) or in the presence of 100 μM cystamine at 4°C for 1 h (E). The ratios represent the intensity of bands of dimer or monomer forms of iNOS normalized against the vehicle control group. Representative experiments in panels A to E were repeated at least three times with similar results. (F to H) Reduction in NO or lipid ROS levels was detected in 5 μM erastin-treated cells in the presence of 100 μM cystamine. After treatment for 8 h, accumulation of NO and ROS was determined following DAF-FM-DA (F, G) or C11-BODIPY (H) labeling coupled with flow cytometry. Scale bar = 20 μm. (F) Images are representative of three independent experiments.

It should be noted that iNOS is catalytically active only in its disulfide-linked homodimer form. Given that PDI is a ubiquitous dithiol/disulfide oxidoreductase in the thioredoxin superfamily, we postulated that PDI might be involved in regulating iNOS dimerization in erastin-treated MDA-MB-231 cells. As expected, using nondenaturing SDS-PAGE and Western blotting, we found that PDI knockdown by siRNA markedly decreased the levels of the dimeric iNOS in both MDA-MB-231 and 786-O cells that were treated with erastin (Fig. 8A and B).

To determine whether PDI can directly catalyze iNOS dimerization, we first analyzed the lysates prepared from MDA-MB-231 cells and iNOS-expressing HEK293 cells, which were incubated with the recombinant PDI protein. The HEK293 cells were employed in this experiment because they did not express the endogenous NOS genes, and they have been widely used in iNOS biochemical characterization and analysis (26, 27). As anticipated, the formation of the active iNOS dimer was increased by addition of the recombinant PDI protein to the cell lysates (Fig. 8C), accompanied by a decrease in the monomer iNOS, which reflects the increased enzymatic conversion of the monomer iNOS to its dimer form. To further corroborate the above observation, we directly incubated PDI with the recombinant human iNOS protein, which was produced using the mammalian and Escherichia coli expression vectors that contain the same human iNOS cDNA. Increased iNOS dimers could be readily detected after addition of PDI to the incubation mixture (Fig. 8D). These results suggest that PDI is an indispensable mediator of iNOS dimerization.

Next, we further examined the role of PDI’s isomerase activity in iNOS dimerization by studying the effect of cystamine, which is an organic disulfide molecule known to selectively inhibit PDI’s isomerase activity through covalent modification of the cysteine residue in its catalytic site (17, 28). Specifically, we examined the direct inhibitory effect of cystamine on PDI-mediated iNOS dimerization *in vitro*. As expected, iNOS dimerization was almost completely abolished by preincubation of the purified PDI protein with cystamine (Fig. 8E). Importantly, we also found that cystamine attenuated not only erastin-induced accumulation of cellular NO (Fig. 8F and G), but also lipid ROS (Fig. 8H), which indicates that the catalytic activation of iNOS was suppressed in MDA-MB-231 cells due to inhibition of PDI by cystamine. Jointly, the results from these experiments showed that PDI’s isomerase activity is responsible for iNOS dimerization in MDA-MB-231 cells.

### PDI is an important mediator of ferroptosis.

**FIG 9:**
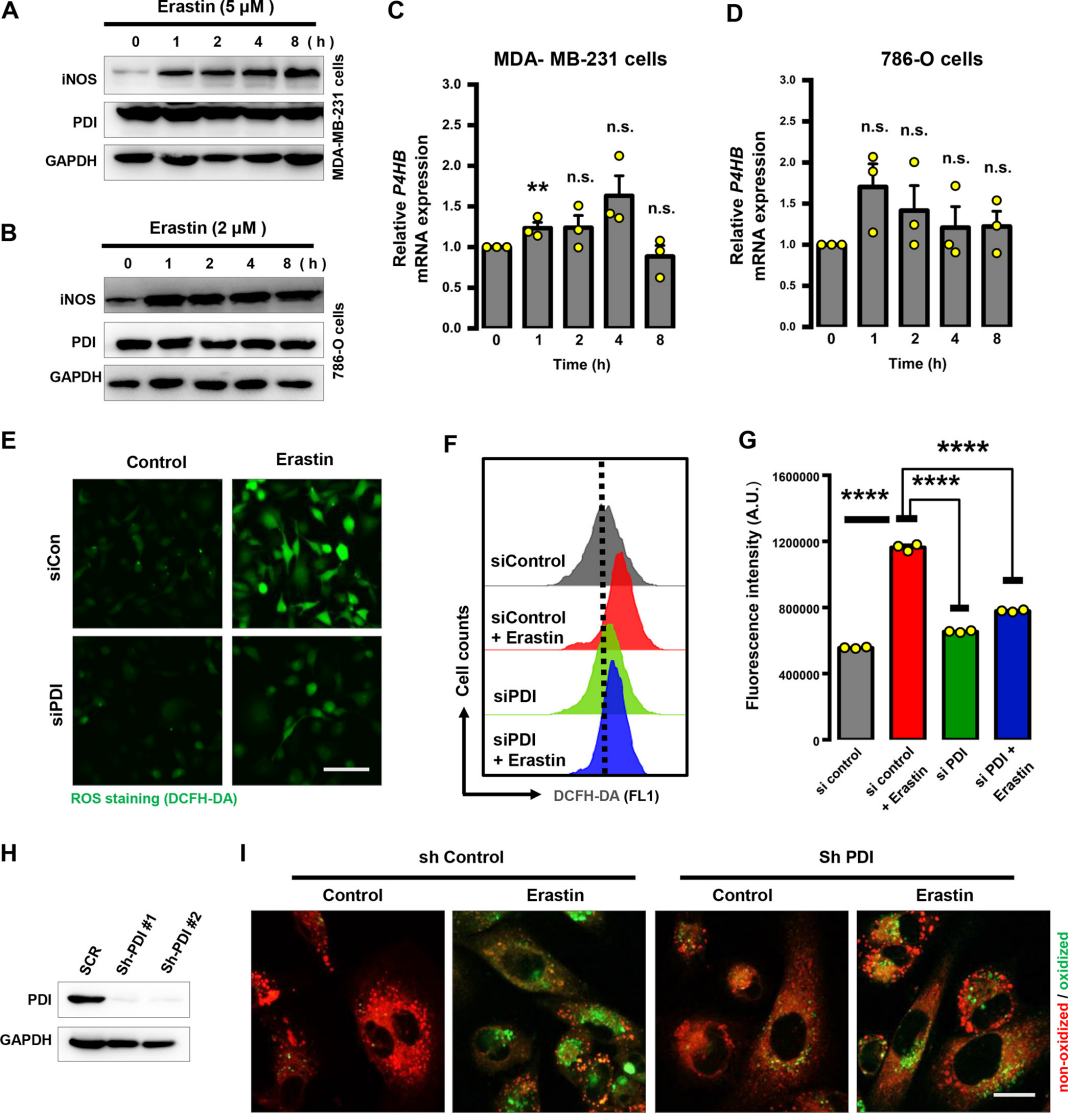
PDI contributed to erastin-induced lipid ROS accumulation. (A to D) Effect of erastin on PDI protein and mRNA levels in MDA-MB-231 and 786-O cells. Protein levels of PDI were measured using Western blot analysis (A, B), and its mRNA levels were analyzed using qRT-PCR (C, D). Data are means ± SD (*n* = 3). **, *P* < 0.01; n.s., not significant. (E to G) PDI knockdown by siRNAs reduced ROS levels. After transfection with siRNAs for 48 h, ROS levels were assessed following DCFH-DA (E) staining coupled with flow cytometry analysis (F, G). Images were taken with a fluorescence microscope (E). Scale bar = 50 μm. Data are means ± SD (*n* = 3). ****, *P* < 0.0001; n.s., not significant. (H) Knockdown of PDI by shRNAs was confirmed by Western blotting. (I) Knockdown of PDI with shRNAs reduced lipid ROS levels, as assessed by C11-BODIPY staining. Scale bar = 20 μm.

Given the critical role of PDI in iNOS activation during ferroptosis, next we sought to determine whether PDI expression is altered by erastin treatment. Western blot results showed that the total PDI protein level was essentially not significantly changed following erastin treatment of MDA-MB-231 and 786-O cells for 8 h (Fig. 9A and B). Quantitative reverse transcription-PCR (qRT-PCR) analysis showed that erastin treatment caused only a very modest increase in PDI mRNA levels initially (at 1 h) in both cell lines, and then PDI mRNA gradually returned to the initial levels at about 8 h (Fig. 9C and D). These results indicate that erastin treatment does not strongly affect the protein and mRNA levels of PDI.

To further substantiate the key role of the PDI in ferroptosis, PDI knockdown was performed. Our results demonstrated that PDI silencing with siRNA or short hairpin RNA (shRNA) effectively prevented erastin-induced accumulation in ferroptosis-associated total (Fig. 9E to G) and lipid (Fig. 9H and I) ROS. These observations resonate with our hypothesis that ferroptosis-associated ROS accumulation likely is dependent on PDI-catalyzed iNOS dimerization.

**FIG 10:**
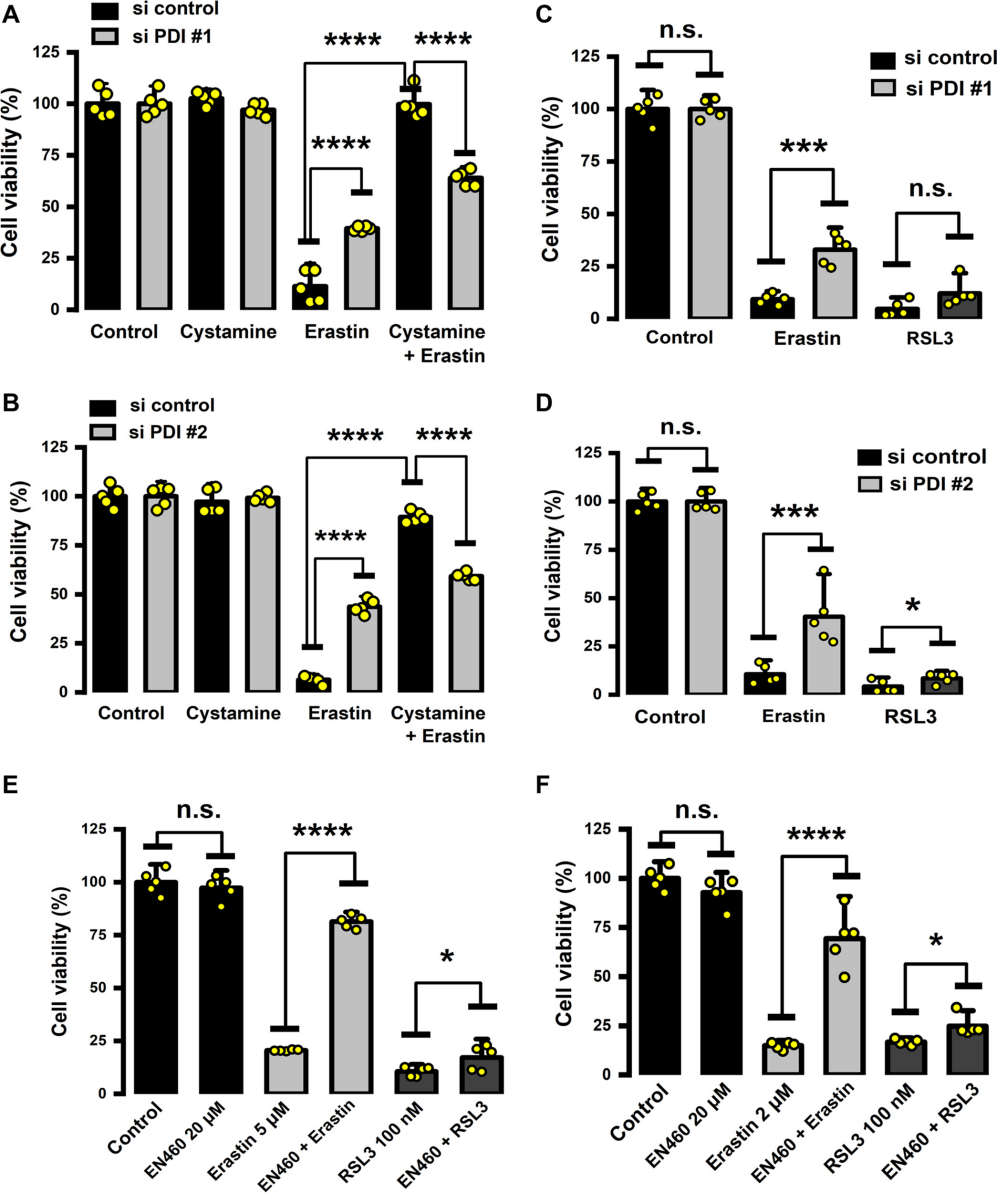
PDI inhibition conferred strong protection against erastin-induced ferroptosis. (A, B) Ferroptotic cell death was partially rescued by PDI inhibition. MDA-MB-231 cells that expressed shPDI or shNC (NC, negative control) were treated with erastin or cystamine alone or in combination for 24 h, and then cell viability was measured by MTT assay. (C, D) 786-O cells were transfected with control siRNA or siRNA to suppress PDI expression. Twenty-four hours after transfection, cells were treated with 2 μM erastin or 100 nM RSL3 for another 24 h, and cell viability was measured by MTT assay. (E, F) Protective effect of the ERO1 inhibitor (EN460) on erastin-induced cell death. MDA-MB-231 (E) and 786-O (F) cells were treated with 20 μM EN460 ± erastin or RSL3 for 24 h, and then the MTT assay was used to determine cell viability. Cell viability change is shown as a percentage of the vehicle control group. Data are means ± SD (*n* = 5). *, *P* < 0.05; **, *P* < 0.01; ***, *P* < 0.001; ****, *P* < 0.0001; n.s., not significant.

Accumulation of the toxic lipid ROS is a hallmark of ferroptosis (2). We found that shRNA-mediated PDI knockdown could effectively suppress erastin-induced ferroptosis in MDA-MB-231 cells (Fig. 10A and B), along with a sharp reduction in lipid ROS accumulation (Fig. 9I), compared to cells transfected with an empty control virus. It is of note that while siRNA-mediated PDI knockdown exerted a strong protection against erastin-induced cell death, which was as expected, no significant protection was observed in cells treated with RSL3, a well-known chemical inducer of ferroptosis through its inhibition of GPX4 (Fig. 10C and D). This observation suggests that PDI activation and its subsequent involvement in mediating chemically induced ferroptosis are closely associated with the conditions of cystine starvation and/or GSH depletion. Consistent with the observed role of PDI in regulating lipid ROS levels and ferroptosis in erastin-treated MDA-MB-231 cells, we found that chemical inhibition of PDI’s catalytic activity by cystamine also afforded a strong protection against erastin-induced cell death (Fig. 10A and B). It is noteworthy that PDI knockdown significantly abrogated the protective effect of cystamine against erastin-induced ferroptosis in these cells, thus demonstrating that PDI is the target that mediates the protective effect of cystamine. In summary, these data showed that erastin-induced cell death in PDI knockdown cells is associated with decreased levels of ROS. Similarly, ROS accumulation and subsequent cell death can be effectively prevented through pharmacological inhibition of PDI. Our data provide evidence for PDI as a potential therapeutic target for protection against chemically induced ferroptosis.

While GSH is a required cofactor for PDI reduction, the endoplasmic reticulum oxidase 1 (ERO1) is involved in PDI oxidation by coupling its disulfide with oxygen reduction to generate hydrogen peroxide (29, 30). The role of ERO1 in PDI-mediated ferroptosis is not known at present. Our results showed that selective inhibition of ERO1 by EN460 (20 μM) exerted a strong protection against erastin-induced ferroptotic cell death in both MDA-MB-231 and 786-O cells (Fig. 10E and F). Notably, when the same concentration of the ERO1 inhibitor EN460 was present, only a very small amount of protection was observed in RSL3-induced ferroptosis, suggesting that the ERO1-PDI pathway plays a more important role in mediating ferroptotic cell death under conditions associated with severe GSH depletion.

## DISCUSSION

While it is known that ROS plays a critical role in the induction of ferroptosis (4, 7, 10, 31), it remains unclear whether NO is involved in ferroptosis-associated ROS accumulation. In the present study, we demonstrated that elevated NO production occurring in the first few hours following exposure of human cancer cells to erastin is an important initial event that subsequently leads to accumulation of the death-inducing cellular lipid ROS. The erastin-induced increase in NO production in the model cell line used in this study is catalyzed by iNOS (but not eNOS), and inhibition of iNOS-mediated NO production can effectively abrogate erastin-induced lipid ROS accumulation and subsequent ferroptosis.

It is known that iNOS is activated through the formation of homodimers, which are stabilized by formation of an intermolecular disulfide bond (25, 26). Our earlier study with an *in vitro* neuronal cell culture model demonstrated that inhibition of the system Xc by high concentrations of extracellular glutamate triggered oxidative cell death that was largely dependent on PDI’s isomerase activity (15). In the present study, we also demonstrated that PDI is directly involved in catalyzing iNOS activation through dimerization in erastin-treated cells, which promotes lipid damage and consequently ferroptosis, based on the following experimental observations. First, iNOS-mediated NO production and the subsequent lipid ROS accumulation were both attenuated in erastin-treated cells by PDI knockdown or silencing. Second, formation of iNOS dimers *in vitro* was increased by addition of PDI to the incubation mixture. Third, PDI’s isomerase activity *in vitro* that catalyzes iNOS dimerization was completely abrogated by preincubation of PDI with cystamine, a known PDI inhibitor (32, 33). Fourth, both iNOS dimerization and its catalytic activation were suppressed by cystamine in erastin-treated cells in culture, which were accompanied by attenuation of erastin-induced accumulation of cellular NO and lipid ROS. Fifth, knockdown or silencing of PDI in live cells greatly diminished the protective effect of cystamine against erastin-induced ferroptotic cell death. Together, these experimental observations revealed that PDI is directly involved in iNOS dimerization, which then contributes to the propagation of lipid ROS and ferroptosis. Given that PDI inhibition confers a strong protection against chemically induced ferroptosis, it is suggested that inhibition of PDI might represent an important potential target for the treatment of ferroptosis-related human diseases.

**FIG 11:**
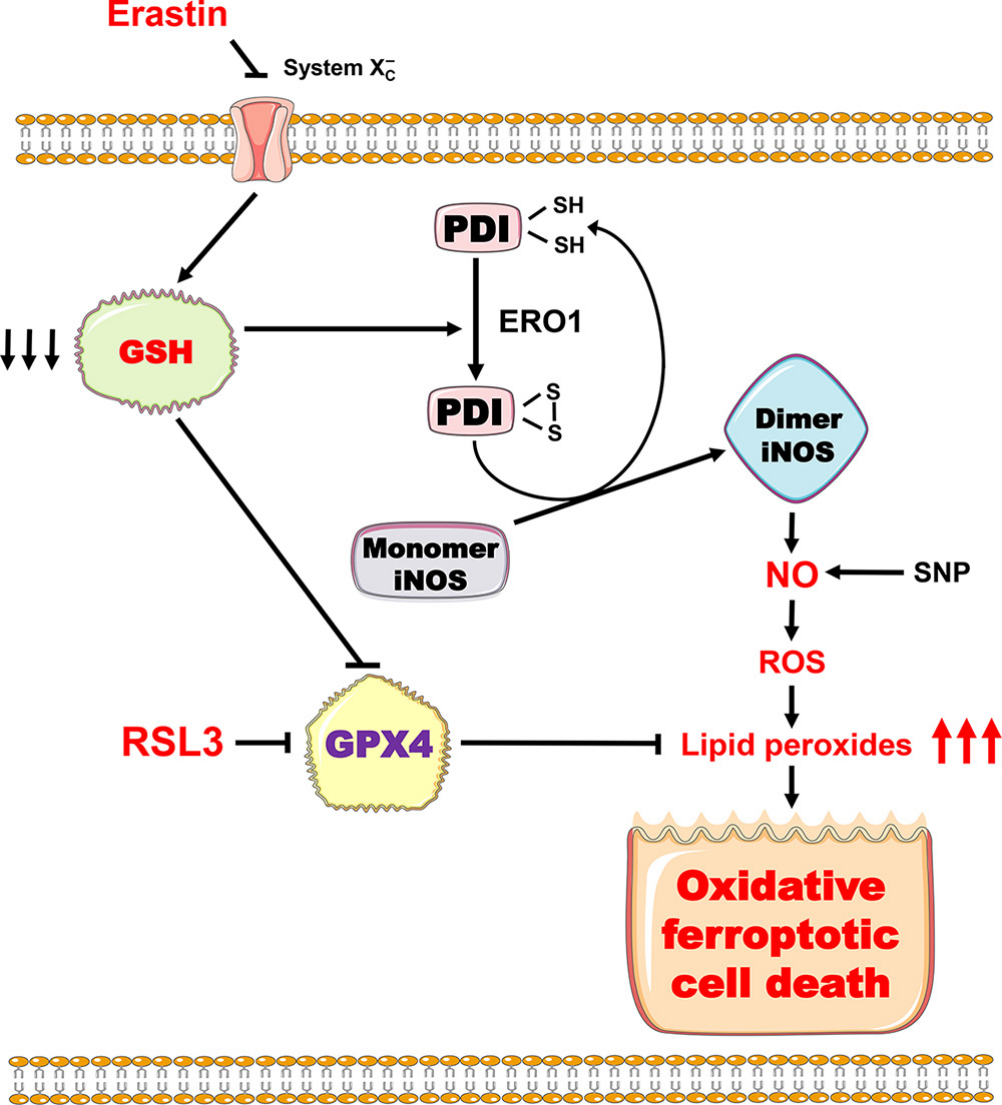
Schematic depiction of the proposed role of PDI in mediating chemically induced ferroptosis.

As depicted in Fig. 11, exposure of cells to erastin is known to inhibit system Xc, which would gradually deplete the cellular GSH (3, 34). GSH depletion would leads to accumulation of the oxidative form of PDI, i.e., the free thiol groups at the catalytic site of PDI are oxidized to form a disulfide bond (28). The oxidative PDI is the active form that can catalyze the conversion of iNOS monomer to its dimer form, and following this reaction, the oxidized PDI is reduced to its reductive state again. ERO1 is an enzyme that aids in the catalytic conversion of the reduced PDI to its oxidative state, and this reaction is favored when GSH is depleted (which is usually accompanied by high GSSG levels) (17, 35, 36). In line with of this mechanistic explanation, we have shown in this study that EN460 a selective ERO1 inhibitor, exerted a strong protection against erastin-induced ferroptotic cell death in both cell lines tested. However, EN460 at the same concentration exerted only a very small amount of protection against RSL3-induced ferroptotic cell death. This intriguing observation appears to be in line with the proposed role of PDI in mediating ferroptotic cell death. It is known that RSL3 induces ferroptosis through its direct inhibition of GPX4, resulting in the buildup of lipid ROS, along with a reduction in cellular GSH levels. Based on earlier observations, reduction in cellular GSH levels during RSL3-induced ferroptotic cell death usually is far less severe than erastin-induced ferroptosis (4, 37). Accordingly, it is expected that when the cellular GSH level is not adequately depleted, the ERO1-mediated PDI oxidation would be significantly reduced, resulting in a greater fraction of PDI staying in its reductive state. Under such conditions, pharmacological inhibition of ERO1 may not be able to elicit a strong protection. While this mechanistic explanation is highly plausible, more experimental studies are needed in the future to systematically examine the intriguing role of ERO1 in regulating GSH depletion-associated PDI activation (along with iNOS dimerization) and ferroptosis.

Lastly, it is of note that while the results of our present study have demonstrated an important role of PDI in ferroptosis induction in cultured cells, it would be challenging to clearly establish the impact of PDI in ferroptosis animal models as PDI knockout in mice is embryonically lethal (28, 38). In addition, since PDI is involved in many biological functions with different interacting partners, even a conditional knockout of PDI may affect other physiological processes beyond ferroptosis. Therefore, any future ferroptosis-related inferences drawn from *in vivo* experiments on PDI or its inhibitors should be supported by strong correlations detected for multiple ferroptosis-associated biochemical changes.

## MATERIALS AND METHODS

### Chemicals and reagents.

Erastin was purchased from Selleck Chemicals (Houston, TX, USA) and dissolved in dimethyl sulfoxide (DMSO). EN460 (M07515) was bought from Biolab (Beijing, China). Sodium nitroprusside dihydrate (SNP) was obtained from Beyotime Biotechnology (S0015; Shanghai, China). Cystamine, Fer-1, Trolox, *N*-acetylcysteine (NAC), and PDI antibody (P7372) were obtained from Sigma-Aldrich (St. Louis, MO, USA) and dissolved in RNase-free water. Specific antibodies for iNOS (610308) and eNOS (610298) were obtained from Abcam (Cambridge, MA, USA). The goat anti-rabbit horseradish peroxidase (HRP)-conjugated antibody and goat anti-mouse HRP-conjugated antibody were purchased from Santa Cruz Biotechnology (Santa Cruz, CA, USA).

### Cell culture.

The MDA-MB-231 human breast cancer cells and 786-O human renal clear cell carcinoma cells were cultured in RPMI medium or Dulbecco’s modified Eagle’s medium (DMEM), respectively, supplemented with 10% fetal bovine serum (FBS) (Thermo Fisher Scientific, Waltham, MA, USA) and 1% penicillin and streptomycin at 37°C under 5% CO_2_. Experiments were conducted at a cell density of 60 to 80% confluence. The cell line used for experiments was passaged no more than 25 times, authenticated by short tandem repeat (STR) profiling, and routinely tested for mycoplasma.

### Cell viability assay.

The MDA-MB-231 and 786-O cells were seeded into 96-well plates at a density of 3,000 cells/well and treated with different chemicals, as indicated. MTT was added to each well at a final concentration of 0.5 mg/mL, and the mixture was incubated for 3 h (37°C, 5% CO_2_). After incubation, medium was removed, and 100 μL DMSO was added to dissolve the MTT formazan. Absorbance of the MTT formazan was measured using a microplate reader (Biotek, Winooski, VT, USA) at 560 nm. A calcein AM-propidium iodide (PI) kit was also used to double stain live and dead cells according to the manufacturer’s instructions (Solarbio, Beijing, China).

### Analysis of cellular ROS.

Cells were seeded at a density of 2.5 × 10^5^ per well in the 6-well dishes. Cells were incubated with 2 μM C11-BODIPY-581/591 (C11-BODIPY) (D3861; Thermo Fisher Scientific, Waltham, MA, USA) for 15 min or with 5 μM 2′,7′-dichlorodihydrofluorescein diacetate (DCFH-DA) (S0033S; Beyotime Biotechnology, Shanghai, China) for 30 min after receiving different drug treatments. Cells were then digested with trypsin and resuspended in phosphate-buffered saline (PBS). For DCFH-DA and C11-BODIPY staining, the data were collected using the MoFlo XDP cell sorter (Beckman Coulter, Indianapolis, IN, USA) from FL1. A minimum of 10,000 cells per treatment group were analyzed with CytExpert (Beckman Coulter, Brea, USA) or FlowJo (FlowJo, LLC, Ashland, OR, USA) software.

### Fluorescence microscopy.

Cells were seeded at a density of 5 × 10^4^ per well on coverslips placed in the 12-well dishes. Twenty-four hours later, cells were treated as indicated. Coverslips were then washed in Hanks’ balanced salt solution (HBSS) and incubated in HBSS containing 2 μM C11-BODIPY, 5 μM DCFH-DA, or 5 μM 3-amino,4-aminomethyl-2′,7′-difluorescein (DAF-FM-DA) for 20 min at 37°C in 5% CO_2_. Coverslips were then mounted on microscope slides for visualization. Slides were imaged using a confocal laser scanning microscope (LSM 900; Carl Zeiss, Oberkochen, Germany) or using a Nikon Eclipse Ti-U inverted microscope (Nikon, Tokyo, Japan), and images were analyzed with Zen software (Carl Zeiss) or NIS-Elements software (Nikon).

### Assessment of NO production.

Cellular NO production was estimated according to the total nitrite and nitrate levels, using a Griess reagent kit (Thermo Fisher), as described previously (39). The nitrite/nitrate concentration was calculated using a standard curve for sodium nitrite. The assay was carried out according to the manufacturer’s instructions.

### Lentiviral shRNA particle preparation and cell transduction.

The shRNA sequences targeting human PDI (TRC no. TRCN00000290651 and TRCN00000296736; Sigma-Aldrich) and a control scrambled shRNA (Sigma-Aldrich) were separately inserted into the pLKO.1-puro vector. The shRNA-containing pLKO.1 lentiviral plasmid (10878; Addgene, Watertown, MA, USA) was packaged with pMD.2G (12259; Addgene) and psPAX2 (12260; Addgene) in 293T cells with Lipofectamine 3000 (Thermo Fisher) according to the manufacturer’s instructions. Forty-eight hours after transfection, the supernatant containing lentiviral particles was collected (supplemented with 8 μg/mL Polybrene) and used to infect target cells, which were then selected with 1 μg/mL puromycin for at least 5 days. Stable cell lines were collected, and the knockdown efficiency was assessed by Western blotting prior to use in specific experiments.

For siRNA transfection, the cells, after reaching 70 to 80% confluence, were transfected with three mixed siRNAs for PDI using Lipofectamine 3000. Twenty-four hours after seeding of the PDI knockdown cells, the cells were processed for immunoblot analysis of the protein levels, MTT assay of cell viability, and measurement of ROS levels. The siRNA sequences for siPDI are GCAGAGAGGCUGAUGACAUTT (no. 1), CCAAAUACCAGCUCGACAATT (no. 2), and GAUCAUGGCUCUUGCAUUUTT (no. 3), and the sequences for siNC are AUCCGCGCGAUAGUACGUATT (no. 1), UUACGCGUAGCGUAAUACGTT (no. 2), and UAUUCGCGCGUAUAGCGGUTT (no. 3).

The control siRNA and iNOS-specific siRNAs were purchased from Invitrogen. The siRNA sequences targeting iNOS were as follows: UUUCAAAGACCUCUGGAUCUUGACC (no. 1) and AGAGUGAGCUGGUAGGUUCCUGUUG (no. 2).

### Protein expression and purification.

The full-length PDI and iNOS were subcloned into the pET28a expression vector. The expressed proteins were purified using Ni-nitrilotriacetic acid (NTA) agarose beads from Qiagen (Boston, MA, USA), as described previously (40–43). Proteins were separated using SDS-PAGE and jointly analyzed using Coomassie blue staining and Western blotting.

### qRT-PCR.

Total RNA was prepared using the Universal RNA extraction kit (9767; TaKaRa Bio, Inc., Japan), and cDNA was generated using PrimeScript RT master mix reagent (RR036A; TaKaRa Bio, Inc., Japan) according to the manufacturer’s instructions. Quantitative reverse transcription-PCR (qRT-PCR) was performed using iTaq Universal SYBR green supermix (Bio-Rad Laboratories Shanghai, Ltd., Shanghai, China) to detect the mRNA expression levels of the indicated genes. The expression levels of the target genes were normalized by subtracting the corresponding GAPDH (glyceraldehyde-3-phosphate dehydrogenase) threshold cycle (*C_T_*) value. The following primers were used: P4HB forward (5′-GGCTATCCCACCATCAAGTTC-3′) and reverse (5′-TCACGATGTCATCAGCCTCTC-3′) and GAPDH forward (5′-GGAGCGAGATCCCTCCAAAAT-3′) and reverse (5′-GGCTGTTGTCATACTTCTCATGG-3′).

### Immunoblot analysis.

Cells were lysed on ice for 30 min with radioimmunoprecipitation assay (RIPA) buffer (Cell Signaling Technology, Beverly, MA, USA) containing a protease inhibitor cocktail (Sigma-Aldrich). The protein concentration was measured with a bicinchoninic acid (BCA) assay kit (Thermo Fisher). The proteins of interest were separated by electrophoresis with 6% or 10% agarose gel and transferred to polyvinylidene difluoride (PVDF) membranes. The membranes were incubated in 5% skim milk for 1 h at room temperature and then incubated with primary antibodies diluted in the blocking buffer overnight at the following dilutions: PDI, 1:1,000; GAPDH, 1:1,000; β-actin, 1:1,000; iNOS, 1:1,000; and eNOS, 1:1,000. After three washes, the membranes were incubated at room temperature for 1 h with the goat anti-mouse HRP-conjugated antibody (Bioss, Beijing, China) or goat anti-rabbit HRP-conjugated antibody (Bioss). Protein bands were visualized using an automatic chemiluminescence imaging system (Tanon 5200, Shanghai, China).

### Measurement of iNOS dimerization.

For immunoblot analysis of the dimeric and monomeric forms of iNOS, protein samples were prepared with a nonreducing sample buffer without heating. Proteins were then separated on 6% or 8% SDS-PAGE under nondenaturing conditions and transferred to the PVDF membranes by wet transfer under a constant 300 mA for 2.5 h. Other Western blot analysis steps were the same as described above.

### Measurement of PDI catalytic activity.

Purified PDI protein (1 mg/mL) was incubated with cell lysates or recombinant iNOS protein (1 mg/mL) at 4°C for 1 h. Samples were then mixed with a nonreducing loading buffer and incubated at 37°C for 30 min, and the protein was separated by 6% SDS-PAGE under nondenaturing conditions, as described above.

### Statistical analysis.

All quantitative experiments and data analysis described in this study were repeated at least three times. Data are represented as means ± standard deviations (SD), as indicated. Statistical analysis and comparisons were performed using the GraphPad Prism 7 (GraphPad Software, La Jolla, CA) with Student's *t* test as indicated in the figure legends. Statistical significance is indicated as follows: *, *P* < 0.05; **, *P* < 0.01; ***, *P* < 0.001; and ****, *P* < 0.0001.

### Data availability.

All data are presented in the article.

## References

[1] Dixon SJ, Lemberg KM, Lamprecht MR, Skouta R, Zaitsev EM, Gleason CE, Patel DN, Bauer AJ, Cantley AM, Yang WS, Morrison B, Stockwell BR. 2012. Ferroptosis: an iron-dependent form of nonapoptotic cell death. Cell 149:1060–1072. 10.1016/j.cell.2012.03.042. 22632970 PMC3367386

[2] Dixon SJ, Stockwell BR. 2019. The hallmarks of ferroptosis. Annu Rev Cancer Biol 3:35–54. 10.1146/annurev-cancerbio-030518-055844. PMC12851585 41613499

[3] Stockwell BR, Friedmann Angeli JP, Bayir H, Bush AI, Conrad M, Dixon SJ, Fulda S, Gascón S, Hatzios SK, Kagan VE, Noel K, Jiang X, Linkermann A, Murphy ME, Overholtzer M, Oyagi A, Pagnussat GC, Park J, Ran Q, Rosenfeld CS, Salnikow K, Tang D, Torti FM, Torti SV, Toyokuni S, Woerpel KA, Zhang DD. 2017. Ferroptosis: a regulated cell death nexus linking metabolism, redox biology, and disease. Cell 171:273–285. 10.1016/j.cell.2017.09.021. 28985560 PMC5685180

[4] Stockwell BR, Jiang X. 2020. The chemistry and biology of ferroptosis. Cell Chem Biol 27:365–375. 10.1016/j.chembiol.2020.03.013. 32294465 PMC7204503

[5] Gao M, Yi J, Zhu J, Minikes AM, Monian P, Thompson CB, Jiang X. 2019. Role of mitochondria in ferroptosis. Mol Cell 73:354–363.e3. 10.1016/j.molcel.2018.10.042. 30581146 PMC6338496

[6] Hadian K, Stockwell BR. 2020. SnapShot: ferroptosis. Cell 181:1188–1188.e1. 10.1016/j.cell.2020.04.039. 32470402 PMC8157339

[7] Stockwell BR, Jiang X, Gu W. 2020. Emerging mechanisms and disease relevance of ferroptosis. Trends Cell Biol 30:478–490. 10.1016/j.tcb.2020.02.009. 32413317 PMC7230071

[8] Feng H, Stockwell BR. 2018. Unsolved mysteries: how does lipid peroxidation cause ferroptosis? PLoS Biol 16:e2006203. 10.1371/journal.pbio.2006203. 29795546 PMC5991413

[9] Shimada K, Skouta R, Kaplan A, Yang WS, Hayano M, Dixon SJ, Brown LM, Valenzuela CA, Wolpaw AJ, Stockwell BR. 2016. Global survey of cell death mechanisms reveals metabolic regulation of ferroptosis. Nat Chem Biol 12:497–503. 10.1038/nchembio.2079. 27159577 PMC4920070

[10] Yang WS, Stockwell BR. 2016. Ferroptosis: death by lipid peroxidation. Trends Cell Biol 26:165–176. 10.1016/j.tcb.2015.10.014. 26653790 PMC4764384

[11] Yang WS, SriRamaratnam R, Welsch ME, Shimada K, Skouta R, Viswanathan VS, Cheah JH, Clemons PA, Shamji AF, Clish CB, Brown LM, Girotti AW, Cornish VW, Schreiber SL, Stockwell BR. 2014. Regulation of ferroptotic cancer cell death by GPX4. Cell 156:317–331. 10.1016/j.cell.2013.12.010. 24439385 PMC4076414

[12] Zou Y, Palte MJ, Deik AA, Li H, Eaton JK, Wang W, Tseng Y-Y, Deasy R, Kost-Alimova M, Dančík V, Leshchiner ES, Viswanathan VS, Signoretti S, Choueiri TK, Boehm JS, Wagner BK, Doench JG, Clish CB, Clemons PA, Schreiber SL. 2019. A GPX4-dependent cancer cell state underlies the clear-cell morphology and confers sensitivity to ferroptosis. Nat Commun 10:1617. 10.1038/s41467-019-09277-9. 30962421 PMC6453886

[13] Liu J, Li L, Suo WZ. 2009. HT22 hippocampal neuronal cell line possesses functional cholinergic properties. Life Sci 84:267–271. 10.1016/j.lfs.2008.12.008. 19135458

[14] Park SY, Jung WJ, Kang JS, Kim C-M, Park G, Choi Y-W. 2015. Neuroprotective effects of alpha-iso-cubebene against glutamate-induced damage in the HT22 hippocampal neuronal cell line. Int J Mol Med 35:525–532. 10.3892/ijmm.2014.2031. 25503787

[15] Fukui M, Song J-H, Choi J, Choi HJ, Zhu BT. 2009. Mechanism of glutamate-induced neurotoxicity in HT22 mouse hippocampal cells. Eur J Pharmacol 617:1–11. 10.1016/j.ejphar.2009.06.059. 19580806

[16] Okada K, Fukui M, Zhu BT. 2016. Protein disulfide isomerase mediates glutathione depletion-induced cytotoxicity. Biochem Biophys Res Commun 477:495–502. 10.1016/j.bbrc.2016.06.066. 27317486

[17] Ali Khan H, Mutus B. 2014. Protein disulfide isomerase a multifunctional protein with multiple physiological roles. Front Chem 2:70. 10.3389/fchem.2014.00070. 25207270 PMC4144422

[18] Fu X, Zhu BT. 2009. Human pancreas-specific protein disulfide isomerase homolog (PDIp) is redox-regulated through formation of an inter-subunit disulfide bond. Arch Biochem Biophys 485:1–9. 10.1016/j.abb.2008.12.021. 19150607

[19] Bastos-Aristizabal S, Kozlov G, Gehring K. 2014. Structural insight into the dimerization of human protein disulfide isomerase. Protein Sci 23:618–626. 10.1002/pro.2444. 24549644 PMC4005713

[20] Ben Khalaf N, De Muylder G, Louzir H, McKerrow J, Chenik M. 2012. Leishmania major protein disulfide isomerase as a drug target: enzymatic and functional characterization. Parasitol Res 110:1911–1917. 10.1007/s00436-011-2717-5. 22160278

[21] Yoshioka J. 2015. Thioredoxin superfamily and its effects on cardiac physiology and pathology. Compr Physiol 5:513–530. 10.1002/cphy.c140042. 25880503

[22] Yu M, Gai C, Li Z, Ding D, Zheng J, Zhang W, Lv S, Li W. 2019. Targeted exosome-encapsulated erastin induced ferroptosis in triple negative breast cancer cells. Cancer Sci 110:3173–3182. 10.1111/cas.14181. 31464035 PMC6778638

[23] Li M, Wang X, Lu S, He C, Wang C, Wang L, Wang X, Ge P, Song D. 2020. Erastin triggers autophagic death of breast cancer cells by increasing intracellular iron levels. Oncol Lett 20:57. 10.3892/ol.2020.11918. 32793311 PMC7418505

[24] Cinelli MA, Do HT, Miley GP, Silverman RB. 2020. Inducible nitric oxide synthase: regulation, structure, and inhibition. Med Res Rev 40:158–189. 10.1002/med.21599. 31192483 PMC6908786

[25] Kolodziejski PJ, Koo JS, Eissa NT. 2004. Regulation of inducible nitric oxide synthase by rapid cellular turnover and cotranslational down-regulation by dimerization inhibitors. Proc Natl Acad Sci USA 101:18141–18146. 10.1073/pnas.0406711102. 15601772 PMC539786

[26] Kolodziejski PJ, Rashid MB, Eissa NT. 2003. Intracellular formation of “undisruptable” dimers of inducible nitric oxide synthase. Proc Natl Acad Sci USA 100:14263–14268. 10.1073/pnas.2435290100. 14614131 PMC283580

[27] Alp NJ, Channon KM. 2004. Regulation of endothelial nitric oxide synthase by tetrahydrobiopterin in vascular disease. Arterioscler Thromb Vasc Biol 24:413–420. 10.1161/01.ATV.0000110785.96039.f6. 14656731

[28] Xu S, Sankar S, Neamati N. 2014. Protein disulfide isomerase: a promising target for cancer therapy. Drug Discov Today 19:222–240. 10.1016/j.drudis.2013.10.017. 24184531

[29] Yao Y, Lu Q, Hu Z, Yu Y, Chen Q, Wang QK. 2017. A non-canonical pathway regulates ER stress signaling and blocks ER stress-induced apoptosis and heart failure. Nat Commun 8:133. 10.1038/s41467-017-00171-w. 28743963 PMC5527107

[30] Zito E, Melo EP, Yang Y, Wahlander Å, Neubert TA, Ron D. 2010. Oxidative protein folding by an endoplasmic reticulum-localized peroxiredoxin. Mol Cell 40:787–797. 10.1016/j.molcel.2010.11.010. 21145486 PMC3026605

[31] Hassannia B, Vandenabeele P, Vanden Berghe T. 2019. Targeting ferroptosis to iron out cancer. Cancer Cell 35:830–849. 10.1016/j.ccell.2019.04.002. 31105042

[32] Fujita I, Nobunaga M, Seki T, Kurauchi Y, Hisatsune A, Katsuki H. 2017. Cystamine-mediated inhibition of protein disulfide isomerase triggers aggregation of misfolded orexin-A in the Golgi apparatus and prevents extracellular secretion of orexin-A. Biochem Biophys Res Commun 489:164–170. 10.1016/j.bbrc.2017.05.118. 28549585

[33] Hoffstrom BG, Kaplan A, Letso R, Schmid RS, Turmel GJ, Lo DC, Stockwell BR. 2010. Inhibitors of protein disulfide isomerase suppress apoptosis induced by misfolded proteins. Nat Chem Biol 6:900–906. 10.1038/nchembio.467. 21079601 PMC3018711

[34] Cao JY, Dixon SJ. 2016. Mechanisms of ferroptosis. Cell Mol Life Sci 73:2195–2209. 10.1007/s00018-016-2194-1. 27048822 PMC4887533

[35] Khan MM, Simizu S, Kawatani M, Osada H. 2011. The potential of protein disulfide isomerase as a therapeutic drug target. Oncol Res 19:445–453. 10.3727/096504011x13123323849717. 22715587

[36] Lee E, Lee DH. 2017. Emerging roles of protein disulfide isomerase in cancer. BMB Rep 50:401–410. 10.5483/bmbrep.2017.50.8.107. 28648146 PMC5595169

[37] Tang D, Chen X, Kang R, Kroemer G. 2021. Ferroptosis: molecular mechanisms and health implications. Cell Res 31:107–125. 10.1038/s41422-020-00441-1. 33268902 PMC8026611

[38] Parakh S, Atkin JD. 2015. Novel roles for protein disulphide isomerase in disease states: a double edged sword? Front Cell Dev Biol 3:30. 26052512 10.3389/fcell.2015.00030 PMC4439577

[39] Hoshiyama M, Li B, Yao J, Harada T, Morioka T, Oite T. 2003. Effect of high glucose on nitric oxide production and endothelial nitric oxide synthase protein expression in human glomerular endothelial cells. Nephron Exp Nephrol 95:e62–e68. 10.1159/000073673. 14610325

[40] Nagpal L, Haque MM, Saha A, Mukherjee N, Ghosh A, Ranu BC, Stuehr DJ, Panda K. 2013. Mechanism of inducible nitric-oxide synthase dimerization inhibition by novel pyrimidine imidazoles. J Biol Chem 288:19685–19697. 10.1074/jbc.M112.446542. 23696643 PMC3707674

[41] Musial A, Eissa NT. 2001. Inducible nitric-oxide synthase is regulated by the proteasome degradation pathway. J Biol Chem 276:24268–24273. 10.1074/jbc.M100725200. 11312270

[42] Mizunaga T, Katakura Y, Miura T, Maruyama Y. 1990. Purification and characterization of yeast protein disulfide isomerase. J Biochem 108:846–851. 10.1093/oxfordjournals.jbchem.a123291. 2081737

[43] Ali D, Abbady A-Q, Kweider M, Soukkarieh C. 2016. Cloning, expression, purification and characterization of Leishmania tropica PDI-2 protein. Open Life Sci 11:166–176. 10.1515/biol-2016-0022.

